# Three-Level Prediction of Protein Function by Combining Profile-Sequence Search, Profile-Profile Search, and Domain Co-Occurrence Networks

**DOI:** 10.1186/1471-2105-14-S3-S3

**Published:** 2013-02-28

**Authors:** Zheng Wang, Renzhi Cao, Jianlin Cheng

**Affiliations:** 1 Department of Computer Science, University of Missouri, Columbia, Missouri 65211, USA; 2 Institute of Informatics, University of Missouri, Columbia, Missouri 65211, USA; 3 Christopher S. Bond Life Science Center, University of Missouri, Columbia, Missouri 65211, USA

## Abstract

Predicting protein function from sequence is useful for biochemical experiment design, mutagenesis analysis, protein engineering, protein design, biological pathway analysis, drug design, disease diagnosis, and genome annotation as a vast number of protein sequences with unknown function are routinely being generated by DNA, RNA and protein sequencing in the genomic era. However, despite significant progresses in the last several years, the accuracy of protein function prediction still needs to be improved in order to be used effectively in practice, particularly when little or no homology exists between a target protein and proteins with annotated function. Here, we developed a method that integrated profile-sequence alignment, profile-profile alignment, and Domain Co-Occurrence Networks (DCN) to predict protein function at different levels of complexity, ranging from obvious homology, to remote homology, to no homology. We tested the method blindingly in the 2011 Critical Assessment of Function Annotation (CAFA). Our experiments demonstrated that our three-level prediction method effectively increased the recall of function prediction while maintaining a reasonable precision. Particularly, our method can predict function terms defined by the Gene Ontology more accurately than three standard baseline methods in most situations, handle multi-domain proteins naturally, and make *ab initio *function prediction when no homology exists. These results show that our approach can combine complementary strengths of most widely used BLAST-based function prediction methods, rarely used in function prediction but more sensitive profile-profile comparison-based homology detection methods, and non-homology-based domain co-occurrence networks, to effectively extend the power of function prediction from high homology, to low homology, to no homology (*ab initio *cases).

## Background

In the genome era, high-throughput genome, transcriptome, and proteome sequencing is generating an enormous amount of omics data such as gene and protein sequences. Since experimental characterization of these proteins can only be carried out at a selectively small scale, large-scale and high-throughput computational prediction methods are needed to annotate the structures and functions of most of these proteins in order for the biomedical research to effectively utilize this vast resource to study genotype - phenotype relationships. To fill the gap, a variety of computational methods have been developed to predict protein function from protein sequence from different perspectives.

The most commonly used approach to function prediction is based on sequence homology. It uses a sequence comparison/alignment tool to search a target protein sequence against protein sequences with known function annotations in a protein database, and if some homologous hits are found, their function annotations may be transferred to the target protein as predictions. GOtcha [1], OntoBlast [2], and Goblet [3] are tools that use BLAST [4] to search for homologues and then combine the Gene Ontology function terms [5] of homologous hits based on BLAST e-values. PFP [6] uses a more sensitive profile-sequence alignment tool PSI-BLAST [4] to search for remote homologues, and also considers co-occurrence relationships between GO [5] terms in order to improve the sensitivity of prediction.

Phylogenetic relationships between proteins have been proven to be helpful for inferring protein functions [7-9]. Paralogues and orthologues, the two kinds of homologous proteins generated by gene duplication and speciation during evolution, respectively [10], may still share similar functions. Thus, the function of a protein may be inferred from that of its paralogues or orthologues, even though the level of their functional similarity may depend on their evolutionary distance and other factors. As most of phylogenetic-tree based methods assume orthologous proteins are more likely to share similar functions [9], they often generate a phylogenetic tree to elucidate the evolutionary relationships between a target protein and its homologous proteins at first, and then preferably use the functions of its orthologues to infer its function. SIFTER [11], Orthostrapper [12], RIO [13], and AFAWE [14] are typical methods in this category.

Apart from homologous relationships mentioned above, network-based methods exploit other relationships stored in protein networks. Assuming that neighboring proteins interacting in a protein-protein interaction (PPI) network may have similar protein function, some early network-based methods use the functions of the direct (radius-one) neighbors of a target protein in a PPI to infer its function. More advances in this direction include the consideration of statistically enriched functions within neighbors [15,16], the expansion of search from direct neighbors to radius-two and radius-three neighbors [17], and the development of more advanced function inference methods, such as Markov random field [18], random walk [19], and algorithms taking in account the global topology of a network [20-22]. In addition to PPI, Functional Linkage Networks (FLNs) [23] derived from protein interaction, gene expression data, phylogenetic profile, and genetic interaction [22,24-26], have been used to predict protein function. More recently, Domain Co-occurrence Networks (DCN) has been used to predict protein function [16].

In an effort to directly link a protein with its function, machine learning methods, such as Support Vector Machines (SVM) and Artificial Neural Networks, have been developed to predict protein function from scratch. Machine learning methods usually generate features from protein sequence, secondary structure, hydrophobicity, subcellular location, and solvent accessibility, and then use these features as inputs to train a classifier to assign proteins to a number of predefined function categories. ProtFun [27] aims to classify a eukaryotic protein into 14 Gene Ontology (GO) categories and several Enzyme Commission classes. FFPred [28] uses features derived from protein disordered regions and protein sequence profiles with SVM to classify a protein into 300 Gene Ontology classes.

With these approaches developed from a variety of perspectives, protein function prediction remains a challenging, largely unsolved problem, particularly when little information regarding homology and protein interaction is known about a target protein. Both the specificity and sensitivity of function prediction need to be improved in order to reliably make function predictions for most proteins. One consensus in the community is to combine multiple complementary methods and explore and integrate more diverse sources of information to enhance prediction accuracy and broaden the annotation scope [29,30]. In this spirit, we developed a three-level method to cope with the complexity of function prediction at different levels, from high homology, to remote homology, to no homology, and synergistically integrated them into a system that can make function prediction for almost all the target proteins in the 2011 Critical Assessment of Function Annotation (CAFA, http://biofunctionprediction.org/) [31]. At the first level, our method uses PSI-BLAST to search SwissProt [32] for significant homologues of a target protein; at the second level, it applies a sensitive profile-profile alignment tool HHSearch to search against Pfam [33] to gather remote homologues; and at the third level, it detects domains existent in the target protein, and then uses their neighboring domains found in a species-specific Domain Co-occurrence Networks (DCNs) to infer the functions of the target protein, even though there may be no homology between the target protein and its neighboring domains. Our method combining predictions generated at all three levels participated in the 2011 CAFA experiment. In comparison with three base-line methods, our method not only substantially expanded the sensitivity/recall of function prediction by adding profile search and domain network at the top of traditional PSI-BLAST search, but increased the semantic similarity between predicted function terms and true ones according to the Gene Ontology. Another advantage is that our method can readily predict the functions of multi-domain proteins by decomposing a protein into individual domains and aggregating the function predictions of each domain on a Domain Co-occurrence Network.

## Results and discussion

We blindly tested our method in the 2011 Critical Assessment of Function Annotations (CAFA) experiment. In total, CAFA released 48,298 protein targets whose functions were not known to predictors from all around the world to make prediction from Sept. 2010 to Jan., 2011. At the end, 436 of the targets whose functions were later known and deposited into the SwissProt database were used to evaluate the performances of the predictors. In order to gauge the advances in the field, CAFA released the predictions of three baseline methods. The Prior method used the frequency of GO terms in the SwissProt database to select 836 most frequent GO terms for each target as prediction. The BLAST method used BLAST [4] to search a target protein against groups of proteins, where proteins in each group shared a common GO term; and the maximum sequence identify of BLAST alignments with sequences in a group was used as confidence score to rank GO terms for the target protein. The GOtcha method [1] generated GOtcha I-Scores as the sum of the negative logarithm of the e-values resulted from the BLAST search and used them as confidence scores to select GO terms. During the CAFA experiment, our method submitted three predictions or models for each target, which were produced by three different ways of combining predictions of three levels. Predictor 1 mapped predictions of three levels into three intervals of confidence from high to low; predictor 2 weighed predictions of three levels differently from more to less in confidence score calculation; and predictor 3 simply used the frequency of GO terms of all PSI-BLAST hits as confidence scores to select GO terms. The details of these three predictors are described in the Method section. Table 1 lists the minimum, maximum, and average number of GO terms predicted by three baseline methods and our three predictors. The following sub-sections report and discuss the performances of our predictors along with the three baseline predictors using complementary evaluation measures.

**Table 1:** The minimum, maximum, and average number of predictions per target of our three predictors and the three baseline methods.

	Minimum number of predictions	Maximum number of predictions	Average number of predictions
Predictor 1	1	100	73.3
Predictor 2	1	100	56.3
Predictor 3	1	100	57.8
Priors baseline	836	836	836.0
BLAST baseline	1	945	254.1
GOtcha baseline	2	519	135.7

### Precision and recall of top n predictions

We evaluated these methods using precision and recall of GO terms on top *n *predictions ranked by prediction confidence scores, for each *n *in the range [1,20]. One caveat is that multiple GO terms having the same confidence score received the same average rank. For example, if the confidence scores of top three GO term A, B, C are 0.9, 0.8, 0.8, respectively, the rank of them will be 1, 2.5 (i.e. (2+3)/2), 2.5, respectively. The top n groups (GO terms in each group have the same confidence scores) of GO terms were used for evaluation, which makes the actual number of GO terms possibly higher than n. The precision and recall of a protein *i *were calculated as:

$$\text{P}{\text{r}}_{i}=\frac{number\text{\_}of\text{\_}correctly\text{\_}predicted\text{\_}nodes}{number\text{\_}of\text{\_}predicted\text{\_}nodes}$$

$$Rc_{i}=\frac{number\text{\_}of\text{\_}correctly\text{\_}predicted\text{\_}nodes}{number\text{\_}of\text{\_}true\text{\_}nodes}$$

Here, all of the top *n *predicted GO terms and the actual GO terms (determined by experimental methods) of protein *i *were propagated to the root of the Gene Ontology Directed Acyclic Graph (DAG). All the GO term nodes present in the paths of predicted GO terms toward the root were considered predicted GO terms; and all the GO term nodes existing in the paths of the actual GO terms toward the root were considered as true GO terms. The overlapping GO terms/nodes between predicted ones and true ones were considered correctly predicted nodes. For each *n *in [1,20], we calculated the precision and recall for each target protein and averaged them over 436 targets protein as the precision and recall on the data set. Thus, for each method, we got 20 precision-recall pairs to generate a precision-recall curve.

**Figure 1:**
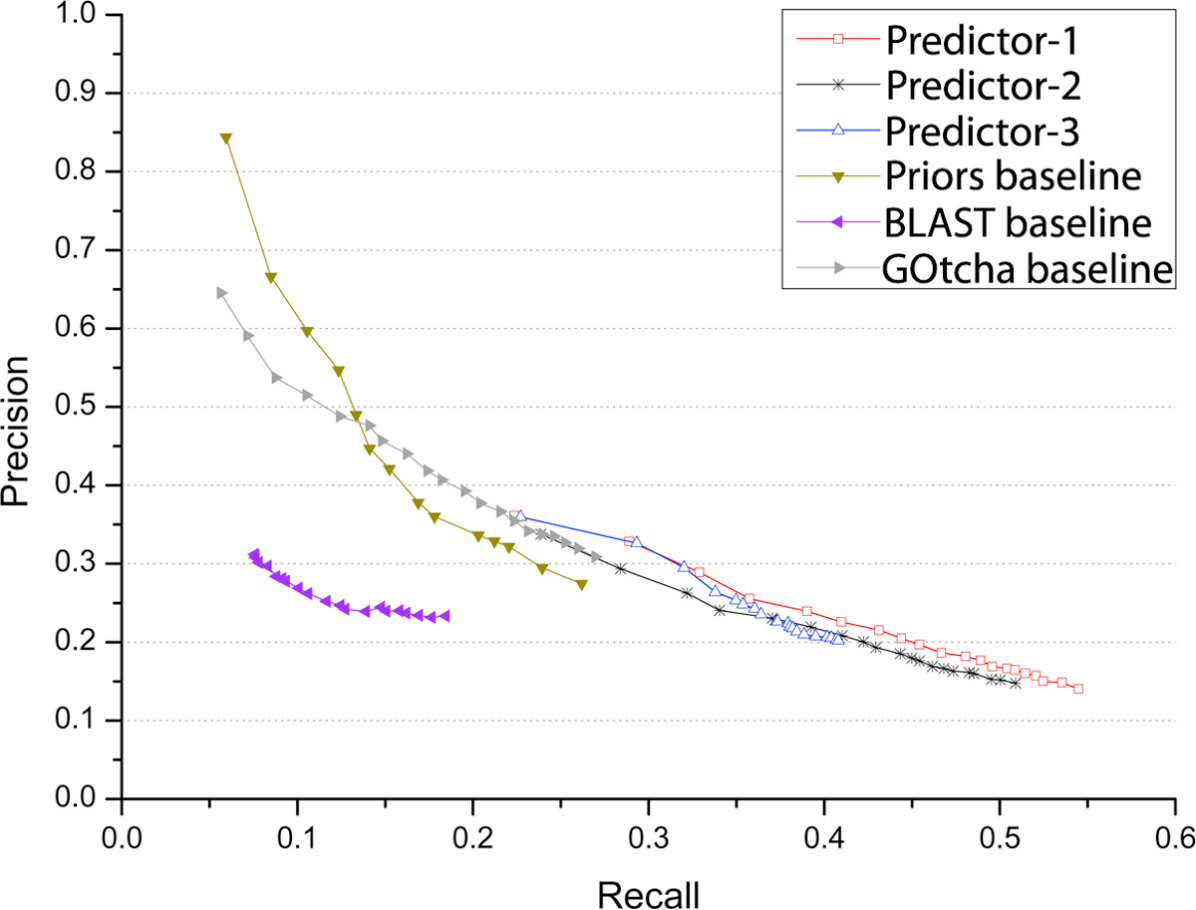
**Precision and recall of our three predictors and three baseline methods when considering top n, 1 < = n < = 20, predictions ranked by confidence scores**.

Figure 1 plots the precision-recall curves of six predictors. The plot shows that our methods tend to have higher recall and lower precision in comparison with the lower recall and higher precision of two baseline methods: Prior and GOtcha. For instance, our best performing predictor 1 can reach a recall value as high as ~0.55, whereas the highest recall value of the baseline methods (i.e. Prior and GOtcha) is at ~0.27. That the baseline methods, particularly the Prior method that selects most frequent GO terms in a general protein function database, can have a high precision but a low recall may be because these methods tend to predict more general GO terms in top n predictions that are closer to the root node of the Gene Ontology, but farther away from the most specific true GO terms. To illustrate this point using an example, we calculated the average depth (number of nodes from a GO term to the root node) of predicted GO terms of the target protein T07719 for the six predictors when recall is at ~ 0.1. The average depth of our predictor 1, 2, and 3, and the Prior, BLAST, and GOtcha is 16.3, 16.3, 18.4, 13.9, 16.1, and 4.8, respectively, which shows that our predictors tried to predict deeper GO terms than the Prior and GOtcha. Although the precision-recall curves of our methods and the three baseline methods largely occupy two different areas in the plot, when their recalls overlap within the range (~0.22, ~0.27), our predictors have higher precisions than the Prior and GOtcha methods at the same recalls. The BLAST baseline method can only have a maximum recall at ~0.18 and a maximum precision at ~0.31, which clearly performed worse than our three methods including our least accurate predictor 3 based on PSI-BLAST search alone. This suggests that PSI-BLAST might work better than BLAST for protein function prediction, even though other factors such as how to rank GO terms based on alignments cannot be ruled out either.

It is also interesting to notice that the best recall value of our predictor 1 (~0.55) is higher than that of predictor 2 (~0.51), indicating that including radius-two domain neighbors in Domain Co-occurrence Network [16] may contribute to the increase of recall since predictor 1 used both radius-one and radius-two domain neighbors to make predictions whereas predictor 2 only used radius-one neighbors (see the Method section for details). That the recall of predictors 1 and 2 are much higher than that of predictor 3 (0.41) demonstrates that profile-profile alignment (HHSearch [34]) and DCN can substantially increase the sensitivity of protein function prediction at the top of the profile-sequence search methods such as PSI-BLAST (e.g. predictor 3).

### Precision and recall under a sliding threshold on confidence scores

We also calculated precisions and recalls according to a sliding threshold scheme, in which only the predictions with confidence scores higher than a threshold value *t *(0 < = *t *< = 1) were selected for evaluation. The predicted and actual GO terms were propagated to the root in the GO DAG as described in the sub-section above. At each threshold, we calculated precision and recall for each protein and used the average precision and recall on all 436 target proteins as the estimated prediction and recall for the threshold. By using the thresholds evenly distributed in the range [0, 1] at step size 0.01, we calculated a series of precision-recall pairs for each of six predictors.

**Figure 2:**
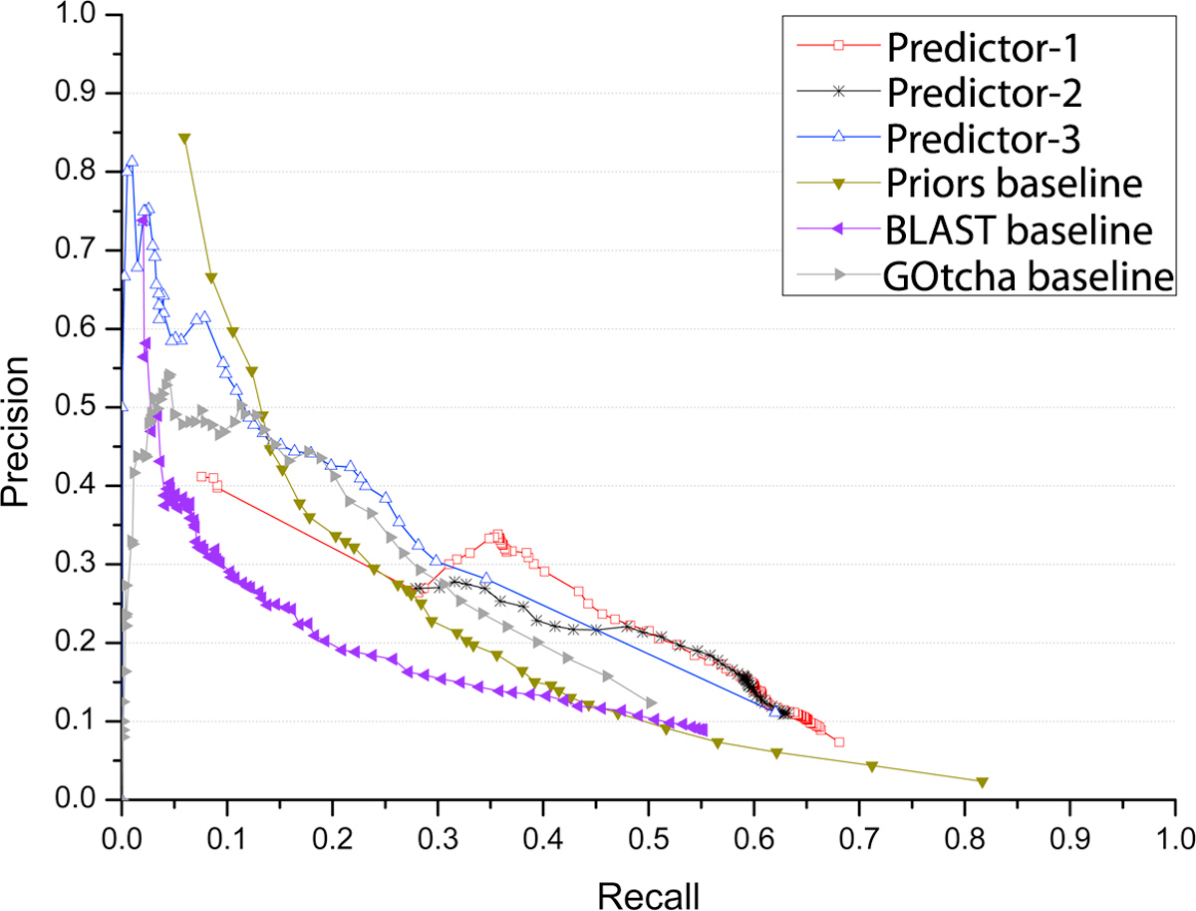
**Precision and recall of our three predictors and three baseline methods when considering predictions with confidence score above a threshold *t*, 0 < = *t *< = 1**. A number of threshold values evenly distributed in the range [0, 1] at step size 0.01 were used to calculated precisions and recalls.

Figure 2 shows the precision-recall curves of our three predictors and three baseline methods. Because all the predictions with a confidence score above a threshold other than just top 20 predictions are selected for evaluation, all the predictors in Figure 2 can reach a higher recall than those in Figure 1. Particularly, the Prior method can yield the highest recall (0.82) among all predictors because it constantly predicts 836 GO terms for each target protein, which is several times more than all other predictors (Table 1). The other two baseline methods (BLAST and GOtcha) that predict more than twice as many GO terms as our methods have the maximum recall values 0.55 and 0.5 respectively, which are lower than the ones of our three predictors. It is worth noting that our predictor 1 that predicted ~73 GO terms on average delivered the second highest recall ~0.68. When recall is higher than ~0.31, our three predictors have higher precision than all three baseline methods at the same recall, while predictor 1 performed mostly better than or occasionally comparable to predictors 2 and 3. That predictor 1 mostly performed better than predictor 2 suggests that the sequential combination of three levels of predictions generated by PSI-BLAST, HHSearch and DCN is more effective than the weighted combination of these predictions. Another interesting observation is that predictor 3 that simply pooled all PSI-BLAST hits to generate GO term predictions performed better than the baseline BLAST and GOtcha methods throughout the entire recall range. It also worked better than the baseline Prior method when recall is > ~0.15, whereas the latter yielded better precision than all other methods when the recall is low (< ~0.15). The better performance of the Prior method in the low recall range may be explained by that its highly common GO term predictions were largely correct, but too far away from the specific GO function of target proteins. Thus its highest precision (~0.85) may serve as an upper limit that current function prediction methods can aim to achieve. Table 2 shows the break-even values of our predictors and the three baseline methods when precision equals to recall. According to this criteria, our methods performed better than the baseline methods, while predictor 1 yielded the highest break-even value 0.306.

**Table 2:** The break-even values between precision and recall (i.e., when precision = recall) of the six predictors.

	Threshold	Precision	Recall	Average
Predictor 1	0.90	0.300	0.311	0.306
Predictor 2	0.81	0.269	0.279	0.274
Predictor 3	0.02	0.304	0.298	0.301
Priors baseline	0.20	0.268	0.270	0.269
BLAST baseline	0.46	0.202	0.193	0.198
GOtcha baseline	0.08	0.293	0.283	0.288

Average values are the averages of precisions and recalls at decision thresholds yielding the closest precision and recall values.

**Figure 3:**
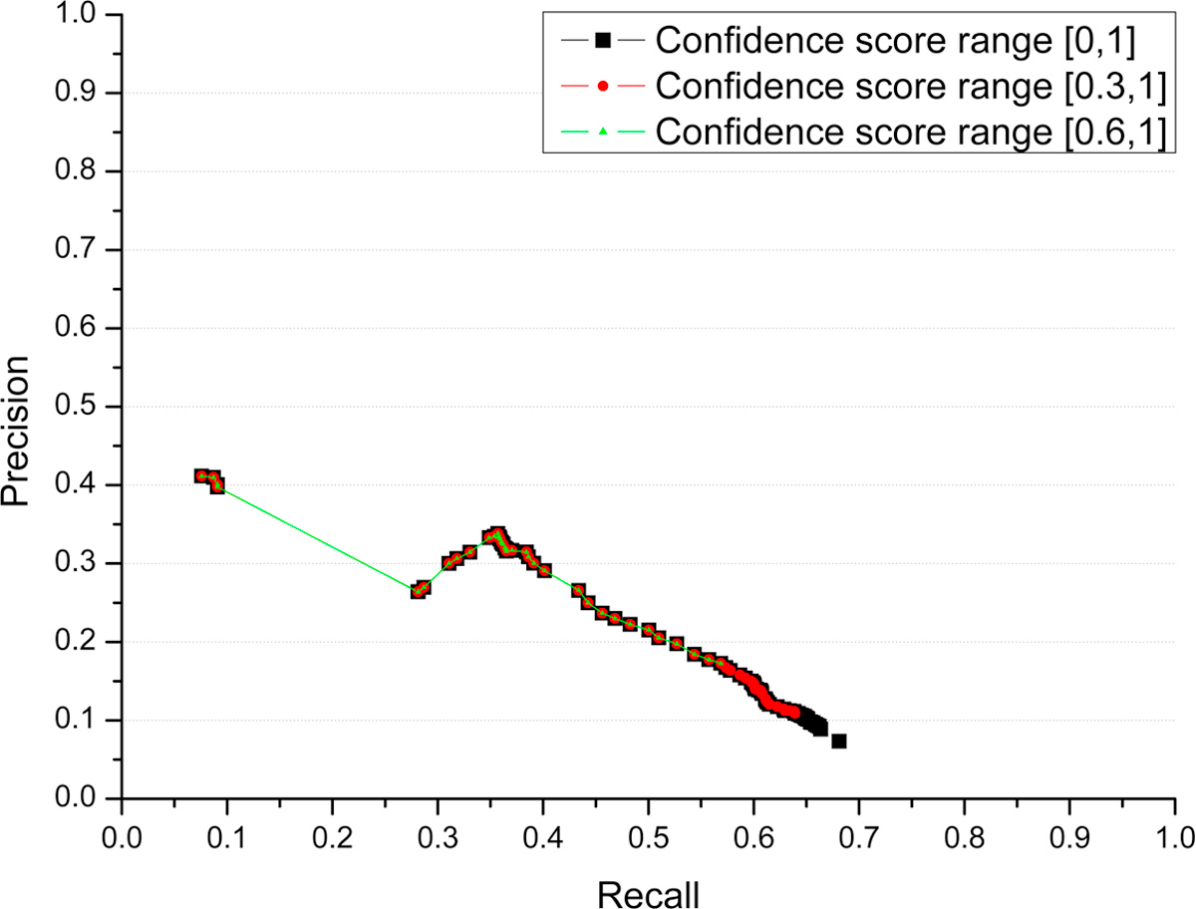
**Precision and recall when progressively considering predictions with confidence score in ranges [0, 1], [0.3, 1], and [0.6, 1] for our predictor 1**. The predictions in the three ranges were predicted by three different methods: PSI-BLAST search, HHSearch search, and Domain Co-Occurrence Networks. Their precision and recall curves were drawn in three different colors, showing the higher level of prediction gradually increased recall at the expense of lower precision.

In order to further analyze the amount of contributions made by profile-sequence alignment (PSI-BLAST), profile-profile alignment (HHSearch), and domain co-occurrence networks (DCN), we plotted a precision-recall curve of predictor 1 in Figure 3 to show how precision and recall changes, when progressively considering predictions resulted from PSI-BLAST search at level 1, from both PSI-BLAST and HHSearch searches at levels 1 and 2, and from all three methods at levels 1, 2 and 3. Figure 3 shows that the profile-profile alignment (HHSearch) extended the recall of profile-sequence alignment (PSI-BLAST) from 0.57 to 0.64, and DCN further increased the recall to 0.69. The results demonstrate that three levels of predictions are complementary and can be combined effectively to increase the sensitivity of protein function prediction. Particularly, the DCN method may contribute valuable function predictions when all homology search methods fail to find useful hits, even though the prediction precision in this *ab initio *situation may be low.

### Evaluations by semantic similarity

**Figure 4:**
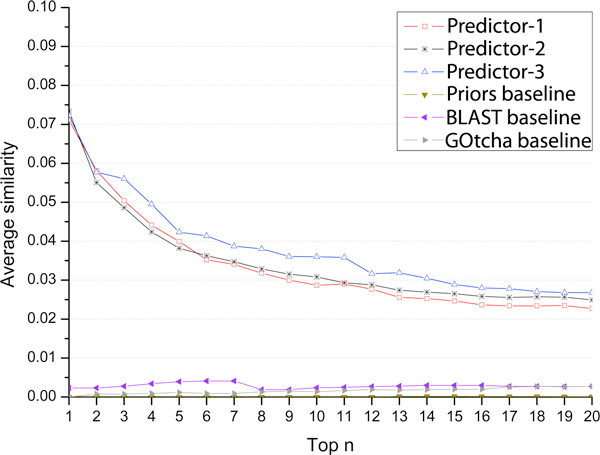
**Average similarity scores of our three predictors and three baseline methods for top 1^st ^.. 20^th ^ranked predictions**.

**Figure 5:**
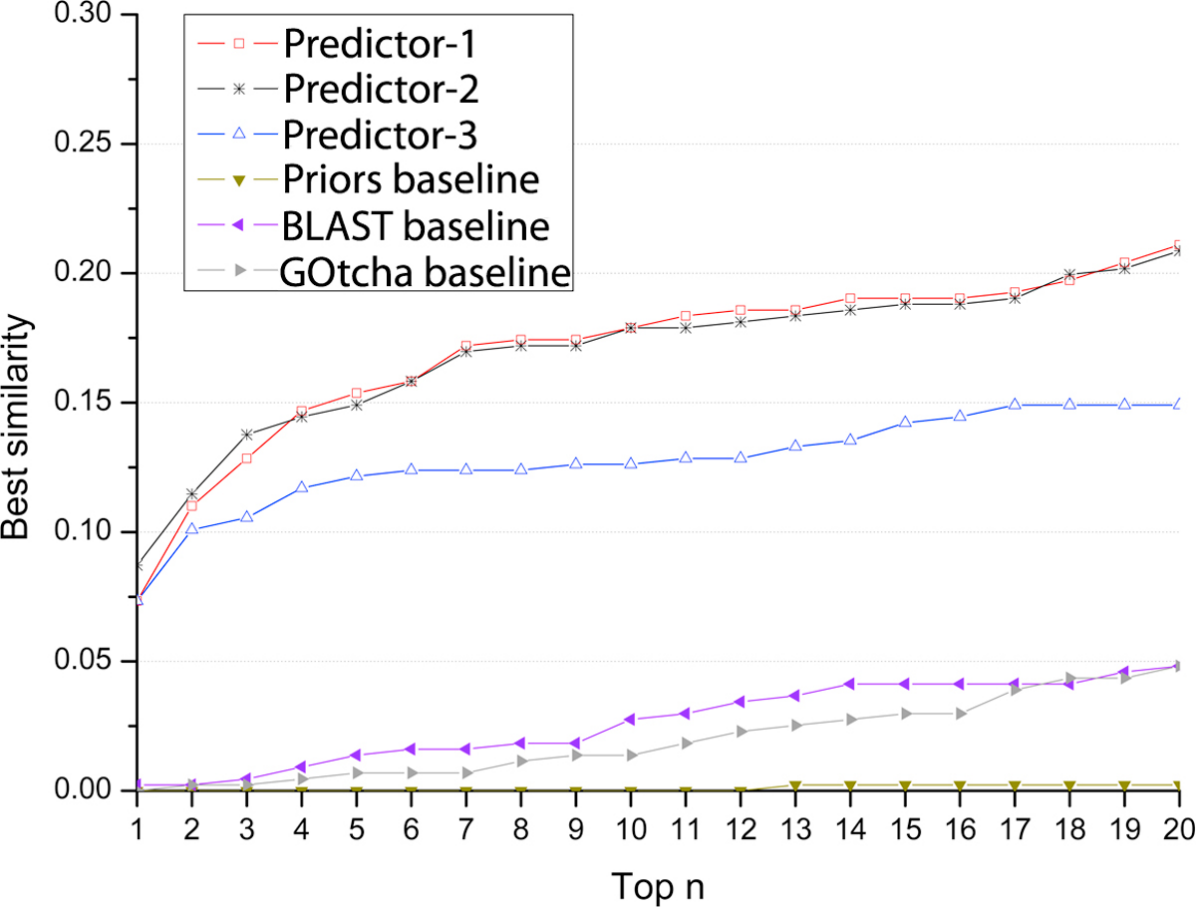
**Best similarity scores of our three predictors and three baseline methods for top 1^st ^.. 20^th ^ranked predictions**.

In addition to precision and recall measures based on the exact match of GO terms, we evaluated these predictors in terms of semantic similarity between true GO terms and predicted GO terms. For two GO terms *g*_1 _and *g*_2_, we obtained their paths *r*_1 _and *r*_2 _to the root of the GO DAG, and calculated the similarity score between *g*_1 _and *g*_2 _as:

$$Sim\left(g_{1},g_{2}\right)=\frac{\theta \left(r_{1}\cap r_{2}\right)}{\text{max}\left(\theta \left(r_{1}\right),\theta \left(r_{2}\right)\right)},$$

where *θ*(*r*_1_) and *θ*(*r*_2_) denotes the number of GO terms of paths *r*_1 _and *r*_2_, respectively. The numerator is the number of common GO terms shared by paths *r*_1 _and *r*_2_. For a target protein, every predicted GO term was compared with each of the true GO terms to calculate similarity scores; and the highest score was considered as the similarity score between a specific predicted GO term and the actual GO terms. Averaging the similarities over all predicted GO terms of a target generated the similarity score for the target. The average similarity scores of all the target protein were used as the prediction similarity score of a predictor. We computed the similarity scores of all predictors for top 1^st^, 2^nd^, ..., 20^th ^ranked, predicted GO terms respectively. It is worth noting that, because the GO terms that have the same confidence scores get the same rank, the set of top 1^st ^.. n^th ^ranked GO terms may actually contain more than n GO terms. The average similarity scores of these predictors were plotted in Figure 4. According to this measure, all our three predictors performed better than three baseline methods. Predictor-3 had higher scores than predictor-1 and predictor-2. It is largely because the latter two more often predicted GO terms with the same confidence scores than the former, resulting in more 1^st ^.. n^th ^ranked GO terms selected for evaluation than the former. In addition to the average similarity score for each target, we also calculated the best similarity score of a target - the highest similarity score among all GO term predictions for the target and averaged the best similarity scores over all the targets for each predictor. Figure 5 shows the best similarity scores of six predictors for top 1-20 predictions, which also demonstrates the better performances of our three predictors compared with the three baseline methods. Predict-1 and predictor-2 performed better than predictor-3 according to this measure.

### An example illustrating the effectiveness of Domain Co-Occurrence Networks for protein function prediction

**Figure 6:**
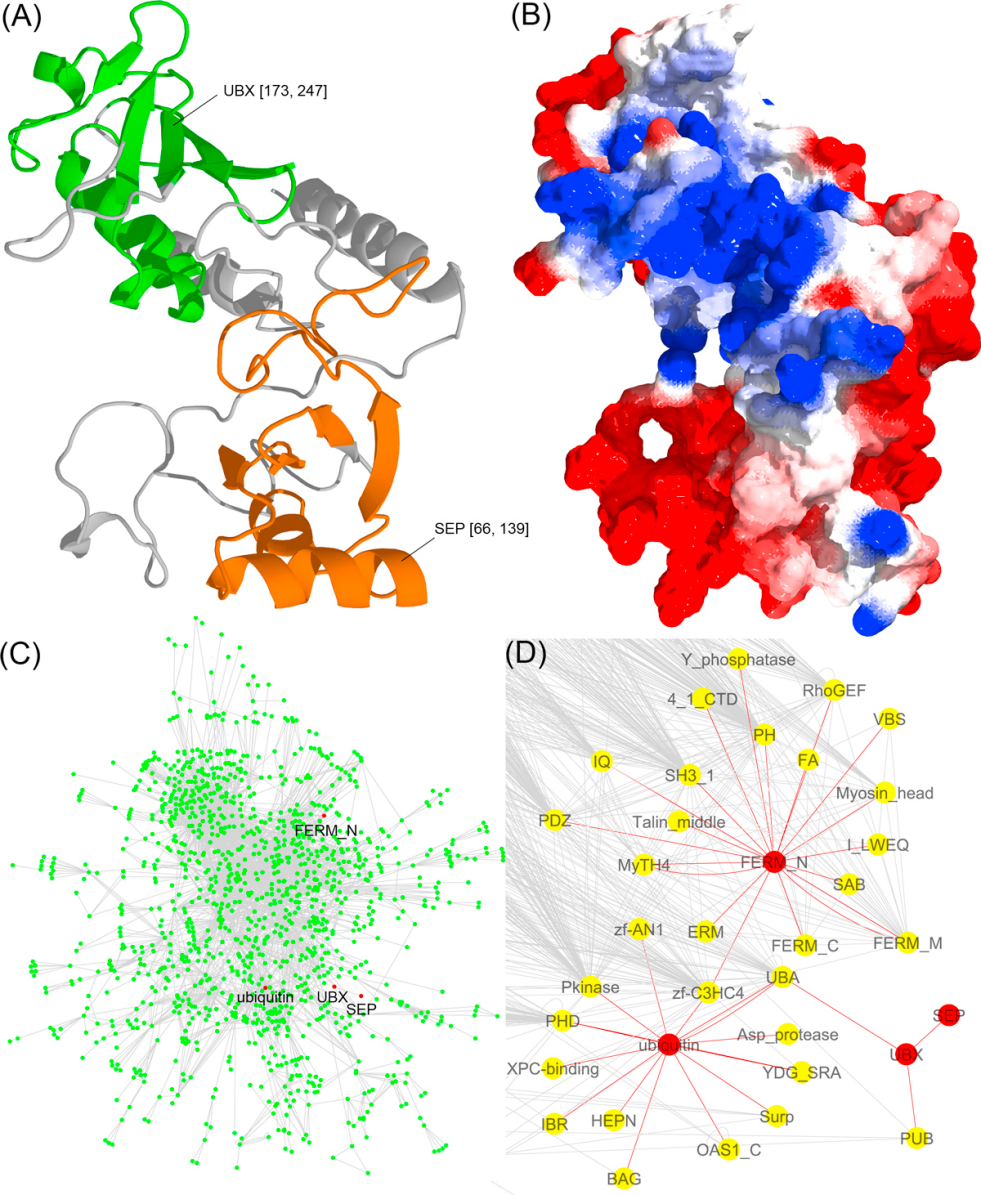
**An example (CAFA target T30248) showing how DCN-based "aggregated neighbor-counting" method works**. (A) Tertiary structure of target protein T30248 predicted by MULTICOM [35] (magenta: domain SEP; green: domain UBX). (B) Electrostatic of protein T30248, generated based on predicted structures in (A) (blue: positive; red: negative). (C) The main Domain Co-occurrence Networks (DCN) of Homo sapiens used to make function prediction. (D) Radius-one neighbor domains of the four domains - ubiquitin, UBX, SEP, and FERM_N - of the target. The GO terms of the neighboring domains were used as predictions for the target. Detailed discussion can be found in "Results and Discussion" section.

We chose an example to illustrate the effectiveness of the DCN function prediction component when both profile-sequence alignment (PSI-BLAST [4]) and profile-profile alignment (HHSearch [34]) cannot make precise predictions. Figure 6 shows how functions were predicted by using Domain Co-occurrence Network (DCN) for target T30248 - a multi-domain protein in *Mus musculus *(house mouse). Figure 6 (A) and 6(B) illustrate the tertiary structure of the protein predicted by MULTICOM [35] and electrostatic potentials (blue: positive charged; red: negative charged), calculated and visualized by DeepView (http://spdbv.vital-it.ch/). In order to make function prediction, the DCN method executed PSI-BLAST to search the target protein against our pre-built protein sequence database containing the proteome of *H. sapiens, S. cerevisiae, C. elegans, D. melanogaster*, 15 plants, and 398 bacteria species (detailed organism names can be found at [16]), and identified the most significant homologous hit - a *H. sapiens *protein (Swiss-Prot ID P68543) with PSI-BLAST e-value 3e-95 and score 348. Then it utilized the DCN of *H. sapiens *(human) (Figure 6 (C)) to make prediction as follows. Firstly it used the profile-profile alignment tool HHSearch [34] to search the target protein against PfamA [33] database, which detected eight domain families with homologous probability > = 0.80: SEP, UBX, Spt20, ubiquitin, UN_NPL4, Cobl, Rad60-SLD, and FERM_N. The four domains (SEP, UBX, ubiquitin, and FERM_N) that existed in the *H. sapiens *proteome were then used as central domains in the DCN of *H. sapiens *to identify domain neighbors. Because domains SEP, UBX, FERM_N do not have GO functional annotations in the Pfam database and the ubiquitin domain only has one general GO term annotation (GO:0006464), directly inferring precise function of the target from these domains was not possible. However, the DCN method was able to use the annotated GO terms of the neighboring domains of these four domains to make function prediction for the target as shown in Figure 6 (D), where red nodes denotes the central domains detected for the target and yellow nodes represents their radius-one neighboring domains. The GO terms of the neighboring domains were aggregated and ranked based on frequency. The top ranked GO terms were used as predictions. The frequencies were used as the confidence scores of the predicted GO terms. Similarly, radius-two neighboring domains (not illustrated) were applied to generate predictions in a similar way.

The DNC method predicted a GO term GO:0006464 (*protein modification process) *using radius-one neighboring domains and predicted GO:0043687 (*post-translational protein modification*), GO: 0051246 (*regulation of protein metabolic process*) and GO: 0008152 (*metabolic process*) using radius-two neighboring domains, which were highly related to the two real GO terms of T30248 (Swiss-Prot ID Q99KJ0.1) - GO:0031396 (*regulation of protein ubiquitination*) and GO:0042176 (*regulation of protein catabolic process*). Moreover, the above-mentioned predicted GO terms all existed in the propagated paths from the actual GO terms to the root in the Gene Ontology Directed Acyclic Graph (DAG). Particularly, the true GO term GO:0042176 (*regulation of protein catabolic process*) and the predicted GO term GO:0051246 (*regulation of protein metabolic process*) had a high semantic similarity score of 0.730 calculated by the tool G-SESAME [36], where 1 indicates exactly the same and 0 completely different. Because the homology-based method did not produce any predictions for T30248, this example demonstrates that the DCN of a species, which may be different from the species of a target protein, can be used to make *de novo *function prediction for the target from scratch. It also shows that the DCN method can readily decompose a multi-domain protein into multiple domains and aggregate function predictions of individual domains as the prediction for the whole protein.

## Conclusions

We designed and developed an automated three-level method to predict protein functions integrating profile-sequence homology search, profile-profile homology search and domain co-occurrence networks. We blindly tested different ways of combining predictions generated at the three levels on a large number of protein targets in the 2011 Critical Assessment of Function Annotation. The results showed that our methods integrating complementary predictions performed mostly better than three standard baseline methods. Our experiments also clearly demonstrated that using profile-profile alignment (HHSearch) and domain co-occurrence networks not only increases the sensitivity of protein function prediction at top of the traditional BLAST- and PSI-BLAST-based homology search methods, but also make it possible to make *ab initio *predictions and handle multi-domain proteins readily.

## Methods

We constructed three protein function predictors (predictor-1, precictor-2, predictor-3), whose predictions were submitted to CAFA as models 1, 2, and 3. The first two predictors combined function predictions derived from profile-sequence PSI-BLAST search, profile-profile HHSearch search, and domain co-occurrence networks using different strategies. The third one used only predictions derived from PSI-BLAST search at the default threshold (i.e. 10).

### Predictor-1

**Figure 7:**
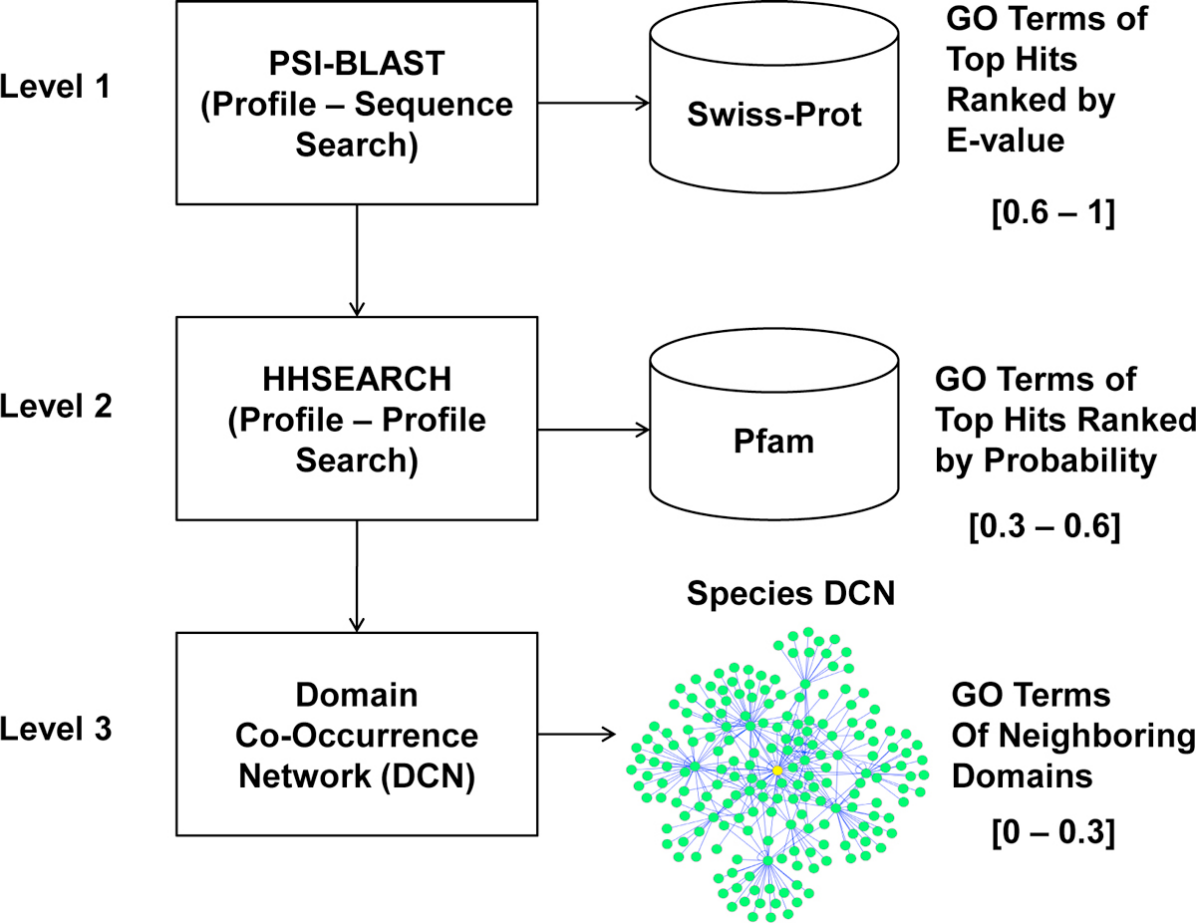
**The architecture of the three-level prediction methodology used in our predictor-1**.

The three-level method integrates profile-sequence alignment, profile-profile alignment, and Domain Co-occurrence Networks (DCNs) as shown in Figure 7. At the first level, PSI-BLAST was executed to search against Swiss-Prot [32]. The protein hits with e-value < = 0.01 were chosen and ranked by e-value. Only the GO terms of the top one hit were included as predictions, whose confidence score *S *was calculated as:

$$S_{PSI-BLAST}=0.6+0.4\times \frac{-\text{lg}\left(e\right)}{200},0.6\leq S_{PSI-BLAST}\leq 1.0,$$

where *e *stands for e-value of the hit assigned by PSI-BLAST. An upper limit of *S_PSI_*_-_*_BLAST _*was set to 1.0. For example, when e-value equals to 0, S is set to 1.0. So S is in the range [0.6, 1]. The second level applied a profile-profile alignment tool HHSearch [34] to detect domains of a target protein. HHSearch generated a hidden Markov model (HMM) for a target protein, which was aligned with the HMM of each Pfam domain family, resulting in a probability score in the range of [0, 100] for each hit. Only the hits with probability score > = 80 were kept, and their GO terms were retrieved from the PfamA database as predictions. The confidence scores of the predicted GO terms were assigned as:

$$S_{HHSearch}=0.3+0.3\times \frac{p}{100},$$

where *p *is the HHSearch probability score from 0 to 100. Thus, the confidence score of GO terms predicted from HHSearch hits is in the range [0.3, 0.6].

The target proteins without predictions made from the first two levels were considered hard cases. For hard cases, we used PSI-BLAST with the default threshold (i.e. 10) to search against both Swiss-Prot and the Gene Ontology database, and additionally applied the DCN-based "aggregated neighbor-counting" method [16] to make predictions. The DCN-based "aggregated neighbor-counting" method ran PSI-BLAST to search a target protein against the pre-built proteome databases to find its most closely related organism. Our database contains the whole-genome protein sequences of *H. sapiens, S. cerevisiae, C. elegans, D. melanogaster*, 15 plant species, and 398 single-chromosome prokaryotic organisms (detailed species names can be found at [16]). The organism whose protein was most similar to the target protein according to the PSI-BLAST search's e-value was considered the most closely related species for the target protein. The pre-constructed DCN of this species was used to make functional predictions for the target. The domain co-occurrence network (DCN) of a species was constructed by the following two steps: (1) each protein sequence of the entire genome was searched against Pfam database [33] using a profile-sequence alignment tool PfamScan to detect the occurrence of any protein domain families in the protein; and every detected domain family was represented as a node in DCN; (2) if two protein domain families co-occurred in one protein, one edge was drawn between them. In this way, a DCN of a species was created, in which a node represents a protein domain family and an edge the co-occurrence relationship between two domain families. Figure 6(C) illustrates the main DCN of *H. sapiens*.

For the domains of the target protein detected by HHSearch at level two, the predictor gathered the GO terms of their radius-one neighboring domains in this DCN as predictions, whose confidence scores was proportional to their occurrence frequencies. If no GO terms could be found in radius-one neighboring domains, it extended to the search to radius-two neighboring domains and made predictions according to the same procedure. The confidence of a predicted GO term was calculated as

$$S_{DCN}=0.3\times f,$$

where *f *is the occurrence-frequency of the GO term - the number of the neighboring domains that have the GO term function divided by the total number of occurrences of all predicted GO terms. Thus, the confidence score of DCN-based predictions is in range (0, 0.3]. The ranges of confidence scores assigned to three levels were chosen according to a benchmarking on 100 proteins randomly selected from Gene Ontology before making predictions for the CAFA targets. We compared the performances of the predictions at each level and set their ranges of confidence scores based on their prediction accuracies on the benchmark proteins from high to low.

### Predictor-2

Predictor-2 used PSI-BLAST to search a target protein against Swiss-Prot with e-value threshold 0.01, applied HHSearch to search the target against PfamA, and employed the DCN-based "aggregated neighbor-counting" method on radius-one neighbors, in order to gather GO terms at all the three levels. The same probability score threshold (> = 80) of HHSearch was used as in Predictor-1. We assigned weights 4, 2, and 1 to a GO term generated by PSI-BLAST, HHSearch, and DCN-based "aggregated neighbor-counting" method, respectively. The weighted frequency of each GO term was calculated and normalized. The normalized score was used as the confidence score of an individual GO term. For proteins without any predictions generated using these three methods, an additional PSI-BLAST search of the protein against Gene Ontology and Swiss-Prot with the default e-value threshold (i.e. 10) was executed in order to gather more hits if possible.

### Predictor 3

Only a PSI-BLAST search against Swiss-Prot with the default threshold (i.e. 10) was performed in predictor 3. All of the PSI-BLAST hits were included to make prediction. The occurrence frequency of a GO term among all hits was used as its confidence score.

## Competing interests

The authors declare that they have no competing interests.

## Authors' contributions

JC conceived and designed the method and the system. ZW implemented the method, built the system, carried out the CAFA experiments. RC, ZW, JC evaluated and analyzed data. ZW, RC, JC wrote the manuscript. All the authors approved the manuscript.

## Declarations

This article has been published as part of *BMC Bioinformatics *Volume 14 Supplement 3, 2013: Proceedings of Automated Function Prediction SIG 2011 featuring the CAFA Challenge: Critical Assessment of Function Annotations. The full contents of the supplement are available online at URL. http://www.biomedcentral.com/bmcbioinformatics/supplements/14/S3.

## References

[B1] Martin D Berriman M Barton G GOtcha: a new method for prediction of protein function assessed by the annotation of seven genomes BMC Bioinformatics 2004 5 1 178 10.1186/1471-2105-5-178 15550167 PMC535938

[B2] Zehetner G OntoBlast function: From sequence similarities directly to potential functional annotations by ontology terms Nucleic Acids Research 2003 31 13 3799 3803 10.1093/nar/gkg555 12824422 PMC168962

[B3] Hennig S Groth D Lehrach H Automated Gene Ontology annotation for anonymous sequence data Nucleic Acids Research 2003 31 13 3712 3715 10.1093/nar/gkg582 12824400 PMC168988

[B4] Altschul S Madden T Schaffer A Zhang J Zhang Z Miller W Lipman D Gapped BLAST and PSI-BLAST: a new generation of protein database search programs Nucleic Acids Research 1997 25 17 3389 3402 10.1093/nar/25.17.3389 9254694 PMC146917

[B5] Ashburner M Ball C Blake J Botstein D Butler H Cherry J Davis A Dolinski K Dwight S Eppig J Gene ontology: tool for the unification of biology Nature Genetics 2000 25 1 25 29 10.1038/75556 10802651 PMC3037419

[B6] Hawkins T Chitale M Luban S Kihara D PFP: Automated prediction of gene ontology functional annotations with confidence scores using protein sequence data Proteins: Structure, Function, and Bioinformatics 2009 74 3 566 582 10.1002/prot.22172 18655063

[B7] Eisen JA A phylogenomic study of the MutS family of proteins Nucleic Acids Research 1998 26 18 4291 4300 10.1093/nar/26.18.4291 9722651 PMC147835

[B8] Goodman M Czelusniak J Moore GW Romero-Herrera A Matsuda G Fitting the gene lineage into its species lineage, a parsimony strategy illustrated by cladograms constructed from globin sequences Systematic Biology 1979 28 2 132 163 10.1093/sysbio/28.2.132

[B9] Sjölander K Phylogenomic inference of protein molecular function: advances and challenges Bioinformatics 2004 20 2 170 179 10.1093/bioinformatics/bth021 14734307

[B10] Sonnhammer ELL Koonin EV Orthology, paralogy and proposed classification for paralog subtypes Trends in Genetics 2002 18 12 619 620 10.1016/S0168-9525(02)02793-2 12446146

[B11] Engelhardt BE Jordan MI Muratore KE Brenner SE Protein molecular function prediction by Bayesian phylogenomics PLoS computational biology 2005 1 5 e45 10.1371/journal.pcbi.0010045 16217548 PMC1246806

[B12] Storm CEV Sonnhammer ELL Automated ortholog inference from phylogenetic trees and calculation of orthology reliability Bioinformatics 2002 18 1 92 99 10.1093/bioinformatics/18.1.92 11836216

[B13] Zmasek C Eddy S RIO: analyzing proteomes by automated phylogenomics using resampled inference of orthologs BMC Bioinformatics 2002 3 1 14 10.1186/1471-2105-3-14 12028595 PMC116988

[B14] Jöcker A Hoffmann F Groscurth A Schoof H Protein function prediction and annotation in an integrated environment powered by web services (AFAWE) Bioinformatics 2008 24 20 2393 2394 10.1093/bioinformatics/btn394 18697771

[B15] Hishigaki H Nakai K Ono T Tanigami A Takagi T Assessment of prediction accuracy of protein function from protein-protein interaction data Yeast 2001 18 6 523 531 10.1002/yea.706 11284008

[B16] Wang Z Zhang XC Le MH Xu D Stacey G Cheng J A Protein Domain Co-Occurrence Network Approach for Predicting Protein Function and Inferring Species Phylogeny PLoS ONE 2011 6 3 e17906 10.1371/journal.pone.0017906 21455299 PMC3063783

[B17] Chua HN Sung WK Wong L Exploiting indirect neighbours and topological weight to predict protein function from protein-protein interactions Bioinformatics 2006 22 13 1623 1630 10.1093/bioinformatics/btl145 16632496

[B18] Deng M Zhang K Mehta S Chen T Sun F Prediction of protein function using protein-protein interaction data Journal of Computational Biology 2003 10 6 947 960 10.1089/106652703322756168 14980019

[B19] Borgwardt K Ong C Schonauer S Vishwanathan S Smola A Kriegel H Protein function prediction via graph kernels Bioinformatics 2005 21 Suppl 1 i47 i56 10.1093/bioinformatics/bti1007 15961493

[B20] Sharan R Ulitsky I Shamir R Network-based prediction of protein function Molecular Systems Biology 2007 3 1 10.1038/msb4100129 PMC1847944 17353930

[B21] Vazquez A Flammini A Maritan A Vespignani A Global protein function prediction in protein-protein interaction networks Nature Biotechnology 2003 21 697 700 10.1038/nbt825 12740586

[B22] Karaoz U Murali T Letovsky S Zheng Y Ding C Cantor CR Kasif S Whole-genome annotation by using evidence integration in functional-linkage networks Proceedings of the National Academy of Sciences of the United States of America 2004 101 9 2888 2893 10.1073/pnas.0307326101 14981259 PMC365715

[B23] Marcotte EM Pellegrini M Thompson MJ Yeates TO Eisenberg D A combined algorithm for genome-wide prediction of protein function Nature 1999 402 6757 83 86 10.1038/47048 10573421

[B24] Linghu B Snitkin E Holloway D Gustafson A Xia Y DeLisi C High-precision high-coverage functional inference from integrated data sources BMC Bioinformatics 2008 9 1 119 10.1186/1471-2105-9-119 18298847 PMC2292694

[B25] Zhao XM Chen L Aihara K Protein function prediction with the shortest path in functional linkage graph and boosting International journal of bioinformatics research and applications 2008 4 4 375 384 10.1504/IJBRA.2008.021175 19008182

[B26] Massjouni N Rivera CG Murali T VIRGO: computational prediction of gene functions Nucleic Acids Research 2006 34 suppl 2 W340 W344 16845022 10.1093/nar/gkl225 PMC1538839

[B27] Jensen L Gupta R Staerfeldt H Brunak S Prediction of human protein function according to Gene Ontology categories Bioinformatics 2003 19 5 635 642 10.1093/bioinformatics/btg036 12651722

[B28] Lobley A Nugent T Orengo C Jones D FFPred: an integrated feature-based function prediction server for vertebrate proteomes Nucleic Acids Research 2008 36 suppl 2 W297 W302 18463141 10.1093/nar/gkn193 PMC2447771

[B29] Hawkins T Chitale M Kihara D New paradigm in protein function prediction for large scale omics analysis Molecular BioSystems 2008 4 3 223 231 10.1039/b718229e 18437265

[B30] Rentzsch R Orengo CA Protein function prediction-the power of multiplicity Trends in biotechnology 2009 27 4 210 219 10.1016/j.tibtech.2009.01.002 19251332

[B31] Radivojac P Clark W Oron TB Schnoes AM Wittkop T Sokolov A Graim K Funk C Verspoor K Ben-Hur A Pandey G Yunes JM Talwakar AS Repo S Souza ML Piovesan D Casadio R Wang Z Cheng J Fang H Gough J Koskinen P Toronen P Nokso-Koivisto J Holm L Cozzetto D Buchan DW Bryson K Jones DT Limaye B Inamdar H Datta A Manjari SK Joshi R Chitale M Kihara D Lisewski AM Erdin S Venner E Lichtarge O Rentzsch R Yang H Romero AE Bhat P Paccanaro A Hamp T Kassner R Seemayer S Vicedo E Schaefer C Achten D Auer F Bohm A Braun T Hecht M Heron M Honigschmid P Hopf T Kaufmann S Kiening M Krompass D Landerer C Mahlich Y Roos M Bjorne J Salakoski T Wong A Shatkay H Wass MN Sternberg MJE Skunca N Supek F Bosnjak M Panov P Dzeroski S Smuc T Kourmpetis YAI van Dijk ADJ ter Braak CJF Zhou Y Gong Q Dong X Tian W Falda M Fontana P Lavezzo E Camillo BD Toppo S Lan L Djuric N Guo Y Vucetic S Bairoch A Linial M Babbitt PC Brenner SE Orengo C Rost B Mooney SD Friedberg I A Large-Scale Evaluation of Computational Protein Function Prediction Nature Methodsaccepted 10.1038/nmeth.2340 PMC3584181 23353650

[B32] Boeckmann B Bairoch A Apweiler R Blatter M Estreicher A Gasteiger E Martin M Michoud K O'Donovan C Phan I The SWISS-PROT protein knowledgebase and its supplement TrEMBL in 2003 Nucleic Acids Research 2003 31 1 365 370 10.1093/nar/gkg095 12520024 PMC165542

[B33] Bateman A Coin L Durbin R Finn R Hollich V Griffiths-Jones S Khanna A Marshall M Moxon S Sonnhammer E The Pfam protein families database Nucleic Acids Research 2004 32 1 276 280 10.1093/nar/gkh121 PMC308855 14681378

[B34] Soding J Biegert A Lupas A The HHpred interactive server for protein homology detection and structure prediction Nucleic Acids Research 2005 33 Web Server W244 W248 10.1093/nar/gki408 15980461 PMC1160169

[B35] Wang Z Eickholt J Cheng J MULTICOM: a multi-level combination approach to protein structure prediction and its assessments in CASP8 Bioinformatics 2010 26 7 882 888 10.1093/bioinformatics/btq058 20150411 PMC2844995

[B36] Du Z Li L Chen C Yu P Wang J G-SESAME: web tools for GO-term-based gene similarity analysis and knowledge discovery Nucleic Acids Research 2009 37 Web Server W345 10.1093/nar/gkp463 19491312 PMC2703883

